# Health-Seeking Behaviors After Acute Respiratory Infection Among Urban and Rural Residents and Different Age Groups: Cross-Sectional Questionnaire Study

**DOI:** 10.2196/97679

**Published:** 2026-08-25

**Authors:** Miao Lai, Ke Yan, Yarong Chen, Dengyu Chen, Sen Xiang, Anqiong Xu, Yao Wang

**Affiliations:** 1 Chengdu Center for Disease Control and Prevention (Chengdu Institute of Health Supervision), No.4, Longxiang Road, Wuhou District, Chengdu, Sichuan, 610041, China, 86 13551220016; 2 Longquanyi District Center for Disease Control and Prevention, Chengdu, Sichuan, China; 3 Jintang County Center for Disease Control and Prevention, Chengdu, Sichuan, China; 4 Qingbaijiang District Center for Disease Control and Prevention, Chengdu, Sichuan, China

**Keywords:** acute respiratory infection, ARI, age groups, health seeking, multistate Markov model, urban-rural

## Abstract

**Background:**

The choice of pathways in health-seeking behavior following acute respiratory infection (ARI) is critical for health care resource allocation, yet traditional logistic regression methods struggle to capture the dynamic evolution of such behaviors.

**Objective:**

This study aimed to quantify the dynamic process of changes in health-seeking behaviors following the onset of ARI and to identify differences in behavioral pathways associated with key factors such as urban-rural status and age.

**Methods:**

This study used a multistate Markov model to quantify transition probabilities and intensities between various health-seeking behaviors, using beta regression models to assess differences in state transition probabilities across urban-rural groups and age groups.

**Results:**

This analysis included 2340 patients with ARI from sampled areas of Chengdu. Subgroup analysis revealed that the rural population was more likely to go directly to a hospital after ARI onset, while the urban population was more likely to purchase medicine directly. Furthermore, there were no significant differences in information seeking and self-monitoring (ISM) use between urban and rural residents after ARI onset. However, following ISM, rural residents were significantly less likely than urban residents to visit a hospital or purchase medicine. Minors and older adults were significantly less likely than adults to engage in ISM after ARI onset. Minors were more likely than adults to directly visit hospitals after ARI onset. After ISM use, adults were more inclined to purchase medicine than minors and older adults, while older adults were more inclined to visit hospitals.

**Conclusions:**

The findings indicated that urban and rural populations, as well as different age groups, exhibited distinct patterns of seeking medical care after ARI onset, and their subsequent behaviors diverged when using ISM. The findings suggested that health interventions should leverage ISM to effectively drive offline actions, implementing targeted strategies tailored to the decision-making characteristics of different populations.

## Introduction

Acute respiratory infection (ARI) represents a major global health issue associated with high morbidity and mortality rates [1]. In 2021, the age-standardized incidence rates for upper respiratory infections and lower respiratory infections were 137,469 per 100,000 and 2853 per 100,000 in China, respectively [2]. After contracting ARI, individuals may engage in various health-seeking behaviors, such as purchasing medicine, searching for information on the internet, and visiting a hospital. Health-seeking behaviors directly impact an individual’s health status, involving not only the process of selecting specific treatment options but also broader patterns of decision-making behavior [3]. Understanding health-seeking behaviors across different groups is crucial. However, significant disparities exist in the allocation of health care resources between urban and rural areas in China, alongside inequities in the use of medical services [4,5], which may lead to differing health-seeking behaviors after falling ill. In addition, individuals across different age groups (minors, adults, and older adults) may exhibit distinct health-seeking behaviors due to variations in immune function and societal roles [6]. Additionally, the use of online resources may influence decisions regarding health-seeking behaviors [7].

Previous studies have predominantly used regression models to examine health-seeking behavior, primarily focusing on factors influencing such behavior [3,8-10]. However, health-seeking behavior is a dynamic and evolving process, with individuals potentially shifting between various behaviors. Traditional regression models struggle to capture this process of state transitions. Multistate Markov (MSM) models can represent multistate processes through transition intensities between different states, dynamically predict transition probabilities between states, and estimate the influence of covariates on transitions [11,12].

The transition between health-seeking behaviors after ARI onset remains unclear. Given Chengdu’s distinct urban-rural integration characteristics, this study selected it as the research site. Using the MSM model, we aimed to quantitatively compare the transition probabilities and pathways of health-seeking behaviors among urban and rural populations, as well as different age groups, following ARI onset. Understanding the health-seeking pathways of different groups may provide data to support the optimization of tiered diagnosis and treatment and the rational allocation of medical resources in Chengdu.

## Methods

### Study Design and Population

A cross-sectional study was conducted in Chengdu using an online questionnaire survey. Participants were recruited using multistage stratified random sampling. Our objective was to recruit patients who had developed ARI within the past 14 days and had recovered by the time of the survey. The questionnaire included information on participants’ sociodemographic characteristics, ARI severity, and health-seeking behaviors following ARI onset. Inclusion criteria were (1) individuals who developed ARI within the past 14 days and had fully recovered prior to the survey, (2) ability to understand the study procedures and sign the informed consent form (for minors and older adults, consent could be signed by their parents, guardians, or children), (3) ability to use the internet and mobile phones to complete the study content and data collection (children and older adults may have their parents, guardians, or children complete the forms on their behalf), and (4) residence in Chengdu for more than 6 months. The exclusion criterion was an incomplete questionnaire response.

Within this cross-sectional framework, we designed a detailed retrospective behavioral recall module in the questionnaire, asking participants to recall and report their main health-seeking behaviors after the onset of ARI (eg, searching for health information via the internet or media, medication purchase, hospital visit, and other health-seeking behaviors) and to record the specific day on which each behavior occurred until recovery. Thus, each participant provided a complete, time-ordered behavioral sequence—a longitudinal trajectory from onset to outcome.

Data were collected from January 16 to 28, 2025, across 6 county-level administrative districts in Chengdu. A total of 19,712 responses were collected during the study period. After applying exclusion criteria, 2340 participants were included in the analysis.

### Definition of ARI and Health-Seeking Behaviors

Given the self-reported nature of this survey, we developed and adopted a standardized operational definition of ARI, drawing on the case definition frameworks from the World Health Organization [13,14], the US Centers for Disease Control and Prevention [15], and the Expert Consensus on the Diagnosis, Treatment, and Infection Control of Acute Respiratory Infections in Adult Outpatient and Emergency Departments [16]. Specifically, ARI was defined as the presence of 1 systemic symptom (eg, fever, chills, headache, or body aches) and 1 respiratory symptom (eg, sore throat, cough, nasal congestion, or runny nose) within 14 days, or the presence of 2 or more respiratory symptoms. For children younger than 2 years or those unable to communicate verbally, ARI was defined as the presence of any one of the following within 14 days: fever (axillary temperature ≥37.0 °C), cough, or respiratory distress.

Health-seeking behaviors refer to the online or offline medical actions individuals take after developing ARI. These behaviors fall into 2 main categories. The first category involves actions that can intervene in disease progression, including self-initiated hospital visits and medication purchases (defined as the self-purchasing of medications). The second category involves digital health monitoring and information retrieval: searching for health information via the internet, media, or AI; consulting online or by telephone; self-checking using wearable devices; or at-home antigen testing. To simplify the Markov model, we consolidated the second category into a single state labeled “information seeking and self-monitoring (ISM).” This consolidation was justified because (1) functionally, all behaviors in this category served the same purpose of information acquisition and self-assessment, rather than representing distinct decision-making stages with different downstream consequences; and (2) analytically, the central question of this study was whether individuals experienced an information-seeking and self-monitoring stage, rather than exhaustively mapping all possible sequences of digital health actions. Within this framework, the first occurrence of any behavior in the second category—along with its timing—was retained and used to define entry into this state. Any subsequent behaviors within the same category, if present, were considered repeated manifestations of the same functional stage and were not treated as separate state transitions to avoid overparameterization. Importantly, the model preserved all transitions between ISM and other behaviors (medication purchase or hospital visit), irrespective of which occurred first.

### ARI Severity

This study used latent class analysis (LCA) to identify the severity of ARI. The number of symptoms and the duration of symptoms were categorized as categorical variables. Combined with absence status, these 3 categorical variables served as manifest variables to construct an LCA model with 1 to 4 latent categories. The optimal number of categories was selected based on the Akaike information criterion (AIC), Bayesian information criterion (BIC), likelihood ratio test (LRT), and minimum category probability. AIC and BIC served as measures of model fit and complexity, where lower values indicated better performance [17]. The LRT was used to assess whether adding a latent category significantly improved model fit compared with a model with one fewer category [18]. The minimum category probability had to exceed 0.10 to prevent model overfitting that could lead to excessive identification of latent classifications within the population [19]. After determining the optimal number of latent categories, each individual was assigned the ARI severity level corresponding to their most probable category membership.

### Statistical Analysis

The baseline characteristics of the participants were summarized using standard descriptive statistics. Categorical variables were presented as frequencies and percentages, while continuous variables were presented as the median and IQR. Where appropriate, comparisons were performed using the Kruskal-Wallis test, Mann-Whitney *U* test, Pearson chi-square test, or Fisher exact test.

An MSM model was constructed to simulate the changes in the sequence of health-seeking behaviors from the onset of ARI to recovery. A first-order time-homogeneous MSM model was used in this study, which assumed that transition intensities between states did not vary over time and depended only on the individual’s current state, not on the historical path by which they arrived at that state. For the MSM models, we defined 4 possible states through which an individual could move (illness, ISM, medicine, and hospital) and one absorbing state (recovery). We defined ARI as entering the “illness” state, with day 0 corresponding to illness onset for each individual. Subsequently, any of the other 3 behaviors might or might not occur. To further simplify the model, we treated “hospital” as the terminal state of the health-seeking pathway and did not track subsequent behaviors after “hospital.” However, transitions from “hospital” to “recovery” (as the absorbing state) were allowed, focusing instead on behavioral changes prior to seeking medical care. MSM models incorporating single covariates (age, sex, urban-rural status, mobile phone use, and severity of ARI) were constructed separately, with significant factors subsequently integrated into a multivariate MSM model. Subgroup analyses of the MSM models containing multiple covariates were performed by age group and urban-rural area. The 14-day transition probabilities and transition intensity matrices between states in the overall and subgroup multivariate MSM models, along with the hazard ratios (HRs) and 95% CIs of the overall multivariate model, were extracted.

The transition probabilities for days 1 through 14 were then entered as the dependent variable into beta regression models (which are appropriate for outcomes bounded within the [0, 1] interval [20]) to examine temporal trends within urban-rural and age subgroups. Three models were fitted: a main effects-only model, a main effects plus time variable model, and a model incorporating the interaction between main effects and time variables. Optimal model selection was based on a comprehensive assessment of AIC values, and coefficients were log-transformed to derive odds ratios (ORs) with 95% CIs.

Two-tailed tests were performed, and *P* value <.05 was considered statistically significant. All statistical analyses were performed using the “*msm*,” “*poLCA*,” and “*betareg*” packages in R software (version 4.5.1; R Foundation for Statistical Computing).

### Ethical Considerations

The study was approved by the Ethics Committee of the Chinese Academy of Medical Sciences and Peking Union Medical College (approval CAMS&PUMC-IEC-2025‐010) and the Chengdu Center for Disease Control and Prevention (Chengdu Institute of Health Supervision; approval 2025003) and was conducted in accordance with the principles of the Declaration of Helsinki. Participants were required to read and agree to the informed consent form online before beginning the questionnaire. No identification of individual participants in any images of the manuscript or appendices is possible.

## Results

### Characteristics of Participants With ARI

**Figure 1.**
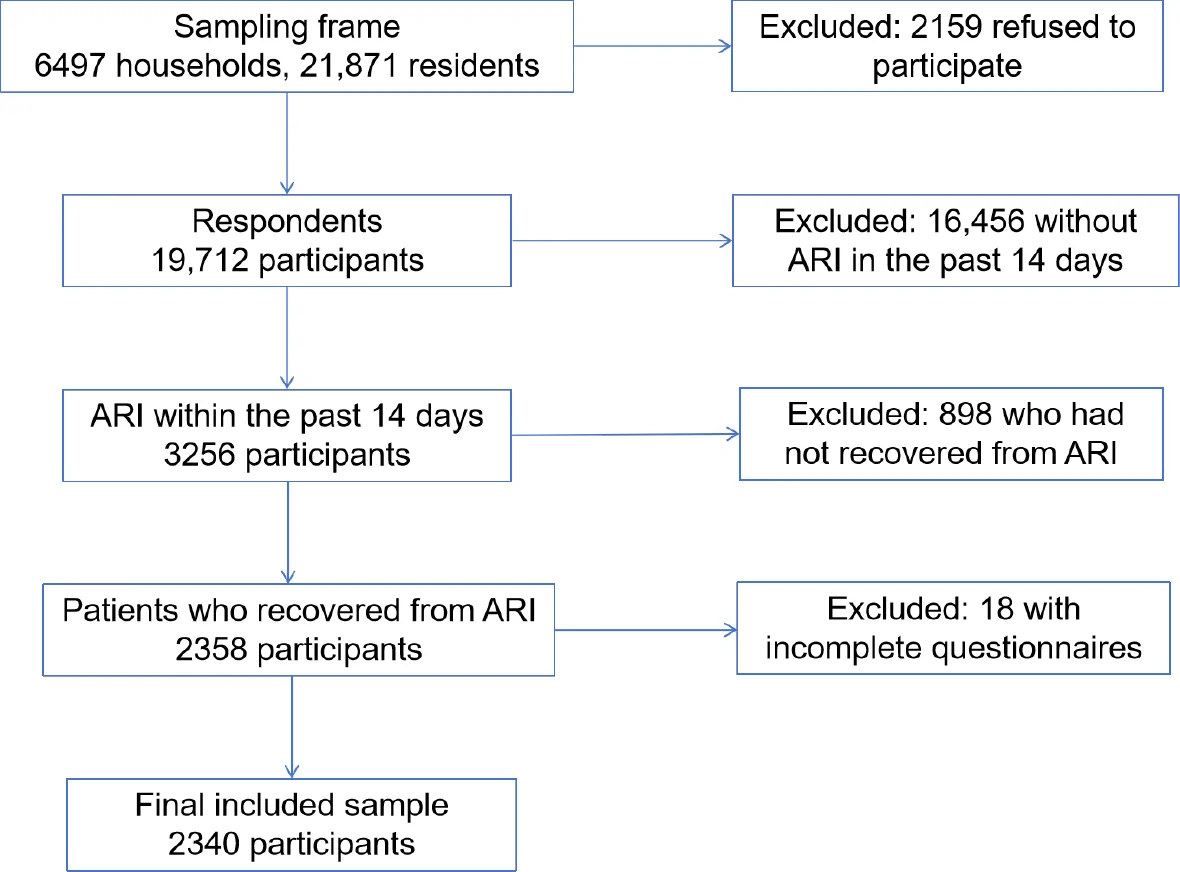
Flowchart of the participant selection process for inclusion in the study analysis. ARI: acute respiratory infection.

The participant selection process is depicted in Figure 1. Of the 2340 participants with ARI included in the analysis, 487 (20.8%), 1592 (68%), and 261 (11.2%) were aged 0 to 17 years, 18 to 59 years, and ≥60 years, respectively, and 1828 (78.1%) and 512 (21.9%) lived in urban and rural areas, respectively (Table 1). On the basis of LCA model fit indices and interpretability (Table S1 in Multimedia Appendix 1), the 2-class model was optimal for ARI severity. This model identified 976 (41.7%) participants in the moderate to severe group and 1364 (58.3%) participants in the mild group. The moderate to severe group was characterized by a high probability of 3 to 5 symptoms (0.60) or ≥6 symptoms (0.36), prolonged symptom duration ≥7 days (0.67), and some absenteeism (0.24). In contrast, the mild group was characterized by a high probability of having only 2 symptoms (0.60), shorter symptom duration of 2 to 6 days (0.62), and minimal absenteeism (0.07). The full conditional probabilities defining these 2 classes were presented in Table S2 in Multimedia Appendix 1. Regarding health-seeking behaviors, significant differences among the 3 age groups were observed in ISM-based behaviors, purchasing medicine, and hospital visits, while differences between urban and rural groups were primarily evident in the latter two behaviors (*P*<.05; Table 1)

**Table 1.** Demographic characteristics and health-seeking behaviors of 2340 patients with acute respiratory infection (ARI) in different age groups and areas of residence.

	Age group				Area of residence		
	0-17 y (n=487)	18-59 y (n=1592)	≥60 y (n=261)	*P* value	Urban (n=1828)	Rural (n=512)	*P* value
Demographics and clinical characteristics							
Age group (y), n (%)							.17
0‐17	—^a^	—	—	—	382 (20.9)	105 (20.5)	
18‐59	—	—	—	—	1254 (68.6)	338 (66)	
≥60	—	—	—	—	192 (10.5)	69 (13.5)	
Area of residence, n (%)				.17			
Urban	382 (78.4)	1254 (78.8)	192 (73.6)		—	—	—
Rural	105 (21.6)	338 (21.2)	69 (26.4)		—	—	—
Sex, n (%)				<.001			.80
Female	242 (49.7)	997 (62.6)	136 (52.1)		1077 (58.9)	298 (58.2)	
Male	245 (50.3)	595 (37.4)	125 (47.9)		751 (41.1)	214 (41.8)	
Mobile phone use, n (%)				<.001			.43
Yes	68 (14)	1561 (98.1)	186 (71.3)		1424 (77.9)	391 (76.4)	
No	419 (86)	31 (1.9)	75 (28.7)		404 (22.1)	121 (23.6)	
ARI severity, n (%)				.001			.92
Mild	248 (50.9)	951 (59.7)	165 (63.2)		1067 (58.4)	297 (58)	
Moderate to severe	239 (49.1)	641 (40.3)	96 (36.8)		761 (41.6)	215 (42)	
Health-seeking behaviors							
Medication purchase, n (%)				<.001			.01
Yes	186 (38.2)	779 (48.9)	106 (40.6)		863 (47.2)	208 (40.6)	
No	301 (61.8)	813 (51.1)	155 (59.4)		965 (52.8)	304 (59.4)	
Time to medication purchase, median (IQR)	1.00 (1.00-2.00)	2.00 (1.00-2.00)	2.00 (1.00-2.00)	.20	2.00 (1.00-2.00)	2.00 (1.00-2.00)	.67
ISM^b^-based behavior, n (%)				<.001			.52
Yes	82 (16.8)	537 (33.7)	31 (11.9)		514 (28.1)	136 (26.6)	
No	405 (83.2)	1055 (66.3)	230 (88.1)		1314 (71.9)	376 (73.4)	
Time to ISM-based behavior, median (IQR)	1.00 (1.00-2.00)	1.00 (1.00-2.00)	1.00 (1.00-2.00)	.89	1.00 (1.00-2.00)	1.00 (1.00-2.00)	.46
Hospital visit, n (%)				<.001			<.001
Yes	243 (49.9)	608 (38.2)	108 (41.4)		708 (38.7)	251 (49)	
No	244 (50.1)	984 (61.8)	153 (58.6)		1120 (61.3)	261 (51)	
Time to hospital visits, median (IQR)	2.00 (1.00-2.00)	2.00 (1.00-3.00)	2.00 (1.00-3.00)	.03	2.00 (1.00-3.00)	2.00 (1.00-2.00)	.16
Time to recovery, median (IQR)	7.00 (4.00-8.00)	6.00 (4.00-8.00)	6.00 (4.00-8.00)	.25	6.00 (4.00-8.00)	6.00 (3.00-7.25)	.42

a Not applicable.

b ISM: information seeking and self-monitoring.

### State Transitions

**Figure 2.**
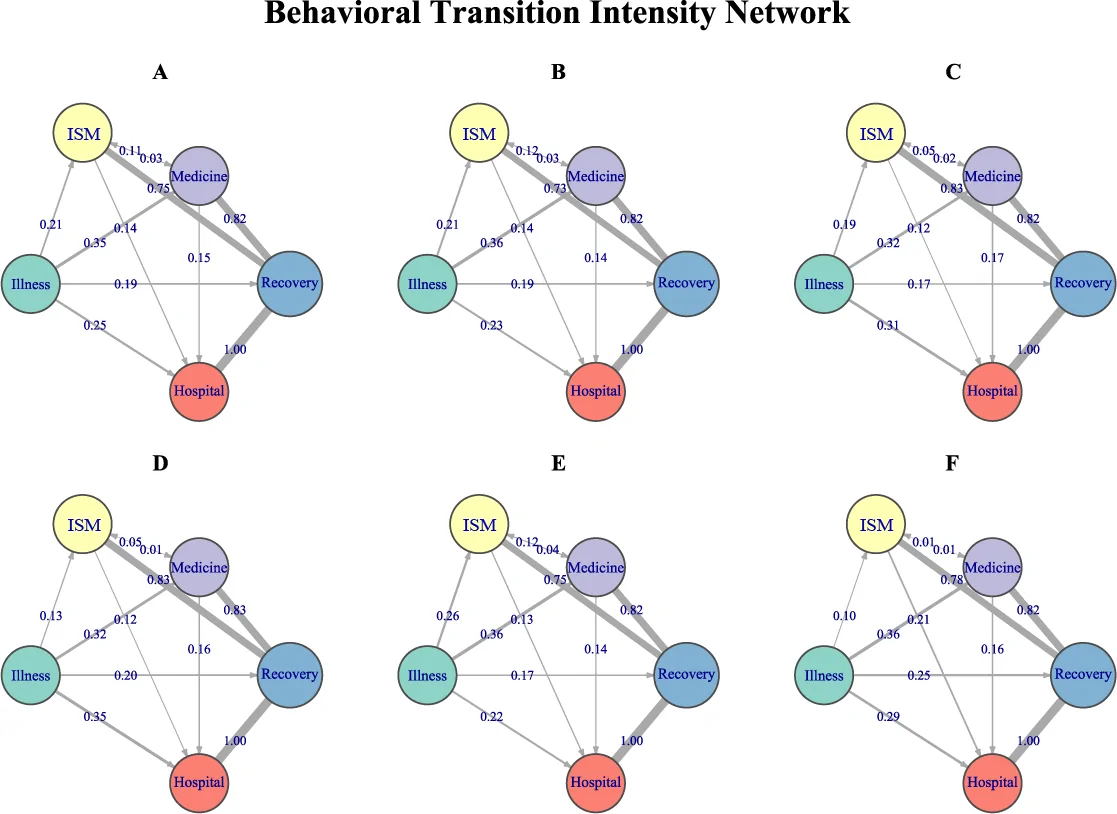
Transition intensities of behaviors among different groups. (A) Total, (B) urban, (C) rural, (D) age group 0‐17 years, (E) age group 18‐59 years, and (F) age group ≥60 years. ISM: information seeking and self-monitoring.

Figure 2 illustrates the intensities of state transitions for the total population, urban and rural populations, and age-specific cohorts calculated using the multivariate MSM model. The effects of covariates on the HR of transitions between different states in the total population are presented in Table S3 in Multimedia Appendix 1. Among the total population (Figure 2A), the intensity from illness to medicine use was highest (0.35, 95% CI 0.34‐0.37), while the intensity from illness to recovery was only 0.19 (95% CI 0.18‐0.20). The intensity of transition from illness to ISM among urban populations (0.21, 95% CI 0.20‐0.23) was close to that of rural populations (0.19, 95% CI 0.16‐0.22). However, the transition intensity from illness to hospital visit was 1.35 times higher among rural populations than among urban populations (0.31 vs 0.23; Figures 2B and 2C). The intensity of transition from ISM to medicine use among urban populations (0.12, 95% CI 0.08‐0.15) was 2.4 times that among rural populations (0.05, 95% CI 0.01‐0.07; Figures 2B and 2C). Similarly, the intensity of transition from ISM to medicine use among individuals aged 18 to 59 years (0.12, 95% CI 0.10‐0.14) was 2.4 times higher than that among those aged 0 to 17 years (0.05, 95% CI 0.01‐0.10) and 12 times higher than that among individuals aged 60 years and older (0.01, 95% CI 0‐0.03; Figures 2D, 2E, and 2F). The intensity of transition from illness to ISM among individuals aged 18 to 59 years (0.26, 95% CI 0.24‐0.27) was 2 times higher than that among those aged 0 to 17 years (0.13, 95% CI 0.10‐0.16) and 2.6 times higher than that among individuals aged 60 years and older (0.10, 95% CI 0.06‐0.13; Figures 2D, 2E, and 2F). The conversion intensity from illness to hospital visit for individuals aged 18 to 59 years (0.22, 95% CI 0.20‐0.23) was 0.63 times that of individuals aged 0 to 17 years (0.35, 95% CI 0.32‐0.39) and 0.76 times that of individuals aged 60 years and older (0.29, 95% CI 0.24‐0.35; Figures 2D, 2E, and 2F).

### State Transition Probabilities Starting From Illness

**Figure 3.**
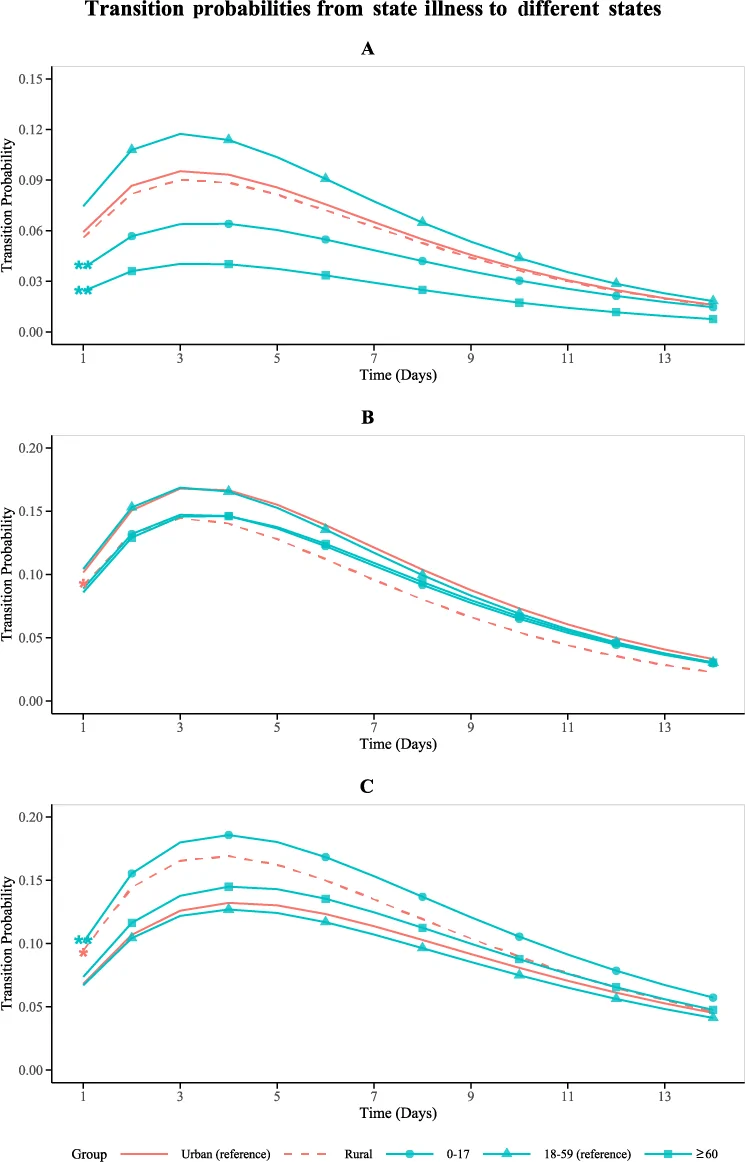
Transition probabilities from the illness state to (A) information seeking and self-monitoring, (B) medicine use, and (C) hospital visit. *Groups with significant differences in beta regression. **Groups remaining significant after false discovery rate correction.

During the observation period from day 1 to day 14, the transition probabilities from the illness state were estimated and plotted in Figure 3. We used a beta regression model to examine transition probabilities over time within urban and rural areas and age subgroups. On the basis of AIC, we selected the optimal model incorporating main effects and time variables, with specific ORs (95% CIs) presented in Table S4 in Multimedia Appendix 1. The beta regression indicated that transition probabilities changed over time (adjusted *P*<.05). The transition probability showed an initial upward trend followed by a decline, with behavioral transitions primarily occurring during the early stages of illness (Figure 3). The transition probability from illness to ISM was significantly lower in both the 0- to 17-year and the ≥60-year age groups compared with the 18- to 59-year age group (both adjusted *P*<.001; Figure 3A). The transition probability from illness to medicine use was significantly lower in the rural group than in the urban group (*P*=.03; Figure 3B). The transition probability from illness to hospital visit was significantly higher in the 0- to 17-year age group than in the 18- to 59-year age group (adjusted *P*<.001), and it was also higher in the rural group than in the urban group (*P*=.048; Figure 3C).

### State Transition Probabilities Starting From ISM

**Figure 4.**
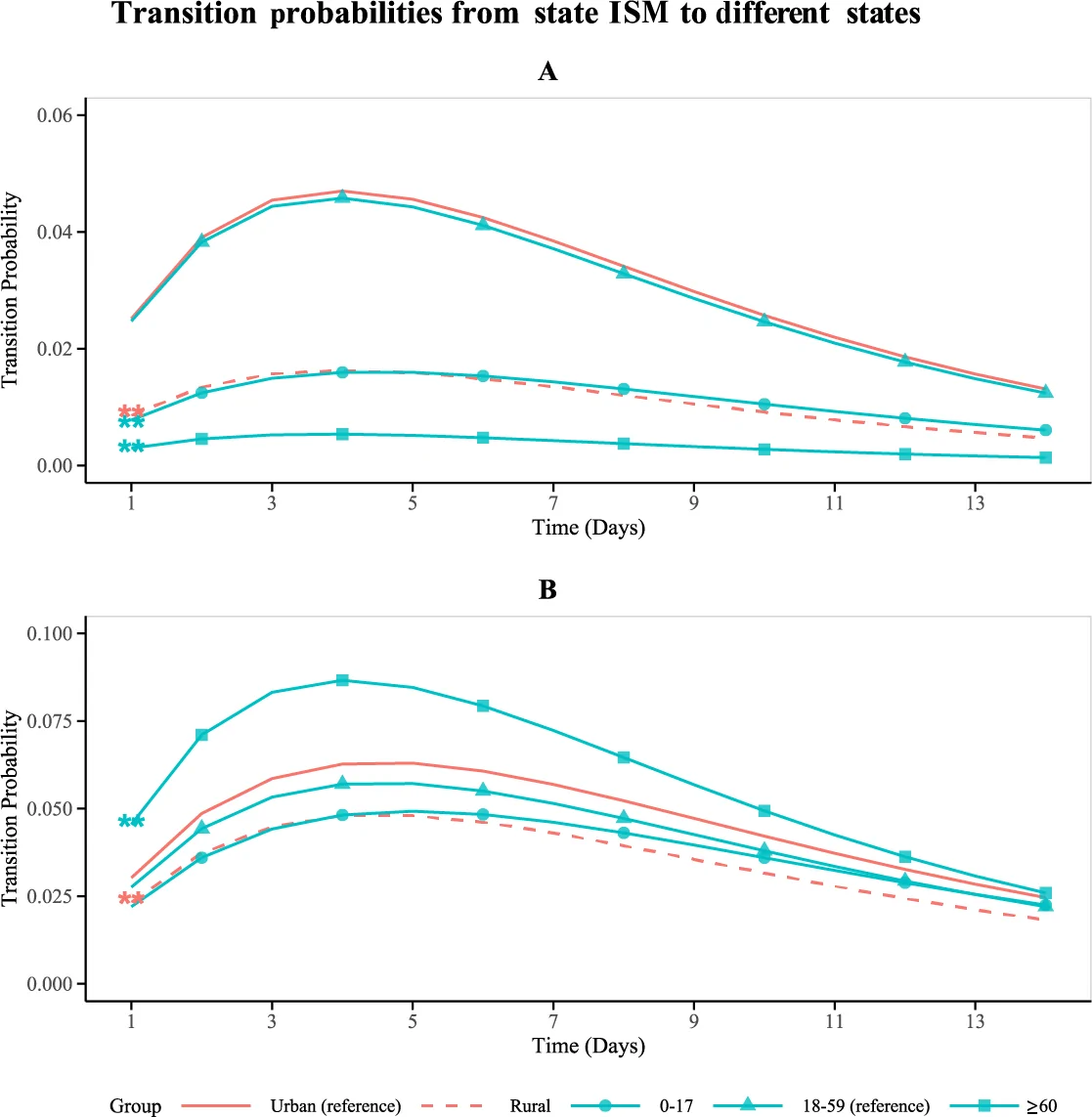
Transition probabilities from the information seeking and self-monitoring (ISM) state to (A) medicine use and (B) hospital visit. *Groups with significant differences in beta regression. **Groups remaining significant after false discovery rate correction.

We estimated the transition probability from ISM over days 1 to 14 and plotted it in Figure 4, also using a beta regression model for analysis. Results indicated that the transition probability changed over time (adjusted *P*<.05). Similar to Figure 3, behavioral transitions primarily occurred during the early stages of the disease (Figure 4). The transition probability from ISM to medicine use was significantly lower in both the 0- to 17-year and the ≥60-year age groups compared with the 18- to 59-year age group (both adjusted *P*<.001), with the lowest probability observed in the ≥60-year group (Figure 4A). In addition, the transition probability was lower in the rural group than in the urban group (adjusted *P*<.001; Figure 4A). The transition probability from ISM to hospital visit was significantly higher in the ≥60-year age group compared with the 18- to 59-year age group (adjusted *P*<.001), but it was also significantly lower in the rural group than in the urban group (adjusted *P*=.02; Figure 4B).

## Discussion

In this cross-sectional study from Chengdu, we observed differences in health-seeking behaviors among various populations after ARI onset. The findings indicated that urban and rural populations exhibited distinct preferences for intervention-related behaviors (medication purchase or hospital visits) following ARI. Furthermore, the study revealed a unique pattern of differentiation: while ISM engagement showed no significant urban-rural disparity, health-seeking behavior choices diverged between urban and rural areas as well as across age groups following ISM engagement. This finding suggested that the role of ISM in health communication was not straightforward but might be modulated by multiple factors including resource accessibility and individual cognition.

The probability of ISM engagement among urban and rural residents was converging. This convergence can be attributed to 2 parallel developments. First, China’s accelerated efforts to expand network coverage in rural areas. To date, 100% of townships and more than 90% of administrative villages have achieved 5G connectivity [21]. Second, the state has lifted restrictions on retail sales of antigen self-test kits [22], and the market for wearable health monitoring devices has expanded rapidly, greatly improving the accessibility of self-monitoring tools [23]. Although ISM engagement rates in urban and rural areas were converging, subsequent behavioral patterns diverged. After engaging in ISM, rural residents were significantly less likely than urban residents to visit hospitals or purchase medicine. This may be attributed to several factors. First, there are significant disparities in functional and critical eHealth literacy (the ability to search for, access, understand, evaluate, communicate, and apply health information within the interplay of individual and societal factors in digital technology [24]) among urban and rural residents [25]. For instance, a study indicated that the proportion of rural students with insufficient eHealth literacy was significantly higher than that of urban students, with 71.4% of this disparity attributed to urban-rural factors and unobserved variables [26]. Additionally, when urban and rural residents translate their online behaviors into offline interventions, they are also influenced by factors such as health care accessibility, economic status, and medical policies [27-29]. It is worth noting that even without ISM access, differences in health-seeking patterns persist between urban and rural residents: rural residents were more likely to go to the hospital after ARI onset, while urban residents were more likely to purchase medicine directly. A survey conducted by Wu [30] across 25 provinces, municipalities, and autonomous regions nationwide yielded the same findings as ours. Wen et al [31] found that self-medication was a common health-seeking behavior, viewed as a low-cost attempt to save money and time. Urban residents, compared with rural residents, have greater access to and convenience in obtaining medications [32]. Despite limitations in the convenience and accessibility of hospitals in rural areas, the influence of distance diminishes when patients have specific demands for health care quality or have more severe conditions [33]. When seeking health care, rural residents may be more inclined to go directly to hospitals rather than attempt self-medication, which carries similar distance and time costs. Additionally, this is attributable to increased income among rural residents, expanded health insurance coverage [34], and differences in employment structures between urban and rural areas [35]. Rural residents engaged in farming possess greater flexibility in managing their time to wait at hospitals.

Minors and older adults were significantly less likely than adults to engage in ISM after ARI onset. For older adults, this reflects a classic gray digital divide [36]. The findings indicated a digital divide existed between those aged 65 years and older and those aged 18 to 64 years in terms of internet access, health-related internet use, health-related social media use, health app use, and the use of wearable electronic health devices [37]. For minors, access to mobile phones is relatively limited, and their roles in ISM engagement and even health-related decisions may be assumed by their guardians. Additionally, minors were more likely than adults to seek direct medical treatment at hospitals after ARI onset. This may be attributed to parents’ fears regarding their children’s illnesses, both nonspecific (persistence or progression of symptoms) and specific (concerns about their child developing a particular disease) [38]. Moreover, differences in social roles and immune status between adults and minors may also lead to different choices. After choosing to engage in ISM, adults were more likely than minors and older adults to purchase medicine, whereas older adults were more inclined to go to hospitals. As mentioned above, adults may opt for more cost-effective approaches to cope. For older adults, whose physical functions decline and who often have underlying health conditions, searching for information online may lead to health-related anxiety, making them more inclined to seek medical care [39,40].

The present study has several limitations. First, questionnaire surveys regarding health-seeking behaviors following ARI may be subject to recall bias. To mitigate this, we restricted recall to the most recent ARI episode within 14 days prior to the survey—a window that balances complete coverage of the typical disease course with short-term recall accuracy—and used anonymous electronic questionnaires to reduce social desirability bias [41]. Nevertheless, recall bias cannot be completely eliminated. Second, to simplify the MSM model, we consolidated various online health ISM behaviors into a single category, which may have resulted in the loss of some detailed path information. However, this consolidation was necessary to ensure model parsimony and estimability. Third, our findings are primarily applicable to patients with ARI who developed the illness within 14 days and had recovered by the time of the survey, systematically excluding those with severe or prolonged illness (>14 days) as well as those in the acute phase who had not yet recovered, thereby limiting generalizability to severe or protracted cases. Fourth, this study adopted a standard first-order Markov assumption and did not consider the effects of state dwell time or preceding states on subsequent transition probabilities. In real-world health-seeking decisions, history-dependent behaviors may exist (eg, whether ISM was used before medication purchase may influence subsequent health-seeking tendencies). Future studies could further explore this using semi-Markov or higher-order Markov models. Furthermore, the generalizability of our findings is constrained by the geographic scope of the study. Data were collected from Chengdu only, and although multistage stratified random sampling was used to ensure representativeness across 6 county-level districts, we did not account for area-level clustering in the analysis. With only 6 districts included, the limited number of clusters precluded stable estimation of random effects. As residents within the same district tend to share similar health care resources and health-seeking patterns, observations may not be fully independent, and standard errors of covariate effects may be underestimated. Therefore, caution is warranted when extrapolating the results to other regions or to the broader Chengdu population. Future studies with broader geographic coverage and a larger number of clusters are needed to further validate these findings and to allow for more robust adjustment of area-level clustering effects.

Overall, our study highlights distinct patterns of health-seeking behaviors among different populations after ARI onset. No difference was found between urban and rural residents in nonintervention behaviors (illness to ISM) after ARI onset, whereas a difference was observed in intervention behaviors (illness to medicine use and illness to hospital visit). Differences across age groups were evident in both types of behaviors. ISM engagement may also influence subsequent intervention behaviors. We hope our findings will provide insights for policymakers, encouraging them to optimize the use of ISM channels and rationally allocate health care resources.

## Supplementary material

10.2196/97679 Multimedia Appendix 1 Model fit indicators in latent class analysis (LCA) for acute respiratory infection (ARI) severity; conditional probabilities of indicators for the 2-class LCA model of ARI severity; effects of covariates on transitions among different states; odds ratio for age groups and urban-rural differences in transition probabilities over time from beta regression.

## Abbreviations

AIC: Akaike information criterion
ARI: acute respiratory infection
BIC: Bayesian information criterion
HR: hazard ratio
ISM: information seeking and self-monitoring
LCA: latent class analysis
LRT: likelihood ratio test
MSM: multistate Markov
OR: odds ratio
