# Supplementary material for: Health-Seeking Behaviors After Acute Respiratory Infection Among Urban and Rural Residents and Different Age Groups: Cross-Sectional Questionnaire Study
Source: J Med Internet Res. 2026 Aug 25;28:e97679. doi: 10.2196/97679 (PMC13505480; doi:10.2196/97679)
Supplement: Supplementary file 1 [file jmir-v28-e97679-s001.doc]

**Appendix**

**Table S1. Model fit indicators in latent class analysis (LCA) for acute respiratory infection (ARI) severity**

| Category | AIC | BIC | *p*-value of LRT | Minimum Category Probability |
| --- | --- | --- | --- | --- |
| 1 | 9744.52 | 9767.55 | / | / |
| 2 | 9597.08 | 9648.90 | <.001 | 0.38 |
| 3 | 9597.50 | 9678.11 | .09 | 0.16 |
| 4 | 9607.50 | 9716.90 | >.99 | 0.13 |

**Table S2. Conditional probabilities of indicators for the 2-class LCA model of ARI severity**

| Indicator | Class 1  Moderate-to-severe  (n = 976, 41.7%) | Class 2  Mild  (n = 1364, 58.3%) |
| --- | --- | --- |
| Symptom count |  |  |
| 2 symptoms | 0.04 | 0.60 |
| 3–5 symptoms | 0.60 | 0.40 |
| ≥6 symptoms | 0.36 | <0.001 |
| Symptom duration |  |  |
| 2–6 days | 0.33 | 0.62 |
| ≥7 days | 0.67 | 0.38 |
| Absenteeism |  |  |
| Yes | 0.24 | 0.07 |
| No | 0.76 | 0.93 |

**Table S3. Effects of covariates on transitions among different states**

| Transition | HR (95% CI) | | | | |
| --- | --- | --- | --- | --- | --- |
|  | Rural vs Urban | 0-17 vs 18-59 | 60+ vs 18-59 | Without mobile phone vs Mobile phone | Moderate-to-Severe vs Mild |
| Illness-ISM | 0.97  (0.80-1.17) | 1.09  (0.75-1.59) | 0.39  (0.27-0.57) | 0.37  (0.25-0.56) | 0.99  (0.85-1.16) |
| Illness-Medicine | 0.93  (0.79-1.08) | 1.19  (0.91-1.56) | 0.87  (0.70-1.07) | 0.65  (0.50-0.85) | 1.08  (0.96-1.23) |
| Illness-Hospital | 1.47  (1.25-1.72) | 1.46  (1.09-1.95) | 1.10  (0.86-1.40) | 1.03  (0.78-1.36) | 1.19  (1.03-1.38) |
| Illness-Recovery | 0.96  (0.79-1.17) | 0.91  (0.65-1.26) | 1.08  (0.83-1.39) | 1.34  (0.99-1.82) | 0.40  (0.33-0.48) |
| ISM-Medicine | 0.57  (0.30-1.08) | 0.47  (0.12-1.83) | 0.77  (0.24-2.53) | 1.76  (0.45-6.87) | 0.89  (0.57-1.39) |
| ISM-Hospital | 0.73  (0.43-1.23) | 1.18  (0.46-3.02) | 1.48  (0.64-3.43) | 0.78  (0.27-2.24) | 1.40  (0.92-2.13) |
| ISM-Recovery | 1.03  (0.84-1.28) | 1.22  (0.80-1.87) | 1.12  (0.74-1.70) | 0.77  (0.48-1.23) | 0.48  (0.40-0.57) |
| Medicine-ISM | 1.19  (0.57-2.51) | 2.63  (0.83-8.26) | 0.48  (0.11-2.07) | 0.15  (0.03-0.71) | 1.07  (0.57-2.00) |
| Medicine-Hospital | 1.14  (0.79-1.66) | 1.02  (0.51-2.05) | 0.98  (0.58-1.66) | 1.12  (0.57-2.22) | 1.29  (0.94-1.77) |
| Medicine-Recovery | 1.03  (0.87-1.22) | 1.05  (0.76-1.45) | 0.99  (0.79-1.25) | 0.93  (0.68-1.28) | 0.52  (0.46-0.60) |
| Hospital-Recovery | 1.04  (0.90-1.20) | 1.09  (0.83-1.42) | 1.04  (0.84-1.29) | 0.91  (0.70-1.17) | 0.61  (0.54-0.69) |

**Table S4. Odds ratio for age groups and urban-rural differences in transition probabilities over time from beta regression**

| Transition | 0-17 vs 18-59 | | 60+ vs 18-59 | | Rural vs Urban | |
| --- | --- | --- | --- | --- | --- | --- |
|  | OR (95%CI) | Adjusted  *P* value | OR (95%CI) | Adjusted  *P* value | OR (95%CI) | Adjusted  *P* value |
| Illness→Illness | 1.12 (0.95-1.33) | .27 | 1.50 (1.27-1.77) | <.001 | 0.92 (0.79-1.07) | .44 |
| Illness→ISM | 0.59 (0.49-0.72) | <.001 | 0.36 (0.29-0.45) | <.001 | 0.95 (0.78-1.16) | .65 |
| Illness→Medicine | 0.89 (0.73-1.10) | .36 | 0.90 (0.73-1.10) | .41 | 0.79 (0.65-0.97) | .10 |
| Illness→Hospital | 1.49 (1.22-1.83) | <.001 | 1.16 (0.94-1.43) | .35 | 1.23 (1.00-1.50) | .14 |
| Illness→Recovery | 0.93 (0.78-1.11) | .45 | 0.99 (0.83-1.18) | .90 | 1.06 (0.89-1.27) | .55 |
| ISM→ISM | 1.42 (1.19-1.69) | <.001 | 0.92 (0.76-1.10) | .43 | 1.16 (0.98-1.37) | .23 |
| ISM→Medicine | 0.38 (0.32-0.46) | <.001 | 0.13 (0.10-0.17) | <.001 | 0.36 (0.29-0.44) | <.001 |
| ISM→Hospital | 0.89 (0.72-1.10) | .36 | 1.42 (1.17-1.73) | <.001 | 0.75 (0.62-0.91) | .02 |
| ISM→Recovery | 0.85 (0.72-1.01) | .14 | 1.14 (0.96-1.35) | .34 | 1.07 (0.90-1.26) | .52 |
| Medicine→ISM | 0.40 (0.33-0.48) | <.001 | 0.29 (0.23-0.36) | <.001 | 0.66 (0.54-0.80) | <.001 |
| Medicine→Medicine | 1.15 (0.97-1.36) | .22 | 0.98 (0.83-1.17) | .90 | 0.91 (0.77-1.08) | .44 |
| Medicine→Hospital | 1.15 (0.95-1.39) | .27 | 1.12 (0.92-1.36) | .38 | 1.10 (0.90-1.33) | .48 |
| Medicine→Recovery | 0.88 (0.74-1.04) | .27 | 1.03 (0.87-1.22) | .89 | 1.07 (0.91-1.27) | .51 |
| Hospital→Hospital | 1.08 (0.91-1.28) | .42 | 0.90 (0.75-1.06) | .35 | 0.91 (0.77-1.08) | .44 |
| Hospital→Recovery | 0.93 (0.78-1.10) | .42 | 1.12 (0.94-1.32) | .35 | 1.10 (0.92-1.30) | .44 |
| Adjusted *P* values were adjusted using false discovery rate correction. | | | | | | |
